# Cocoa flavanols reduce N‐terminal pro‐B‐type natriuretic peptide in patients with chronic heart failure

**DOI:** 10.1002/ehf2.12077

**Published:** 2015-12-08

**Authors:** Rodney De Palma, Imelda Sotto, Elizabeth G. Wood, Noorafza Q. Khan, Jane Butler, Atholl Johnston, Martin T. Rothman, Roger Corder

**Affiliations:** ^1^ Department of Cardiology, The London Chest Hospital Barts Health NHS Trust Bonner Road London E2 9JX UK; ^2^ William Harvey Research Institute, Barts and the London School of Medicine and Dentistry Queen Mary University of London Charterhouse Square London EC1M 6BQ UK

**Keywords:** Heart failure, Endothelial dysfunction, Flavanol, Procyanidin

## Abstract

**Aims:**

Poor prognosis in chronic heart failure (HF) is linked to endothelial dysfunction for which there is no specific treatment currently available. Previous studies have shown reproducible improvements in endothelial function with cocoa flavanols, but the clinical benefit of this effect in chronic HF has yet to be determined. Therefore, the aim of this study was to assess the potential therapeutic value of a high dose of cocoa flavanols in patients with chronic HF, by using reductions in N‐terminal pro‐B‐type natriuretic peptide (NT‐proBNP) as an index of improved cardiac function.

**Methods and results:**

Thirty‐two patients with chronic HF, stable on guideline‐directed medical therapy, were randomized to consume 50 g/day of high‐flavanol dark chocolate (HFDC; 1064 mg of flavanols/day) or low‐flavanol dark chocolate (LFDC; 88 mg of flavanols/day) for 4 weeks and then crossed over to consume the alternative dark chocolate for a further 4 weeks. Twenty‐four patients completed the study. After 4 weeks of HFDC, NT‐proBNP (mean decrease % ± standard deviation) was significantly reduced compared with baseline (−44 ± 69%), LFDC (−33 ± 72%), and follow‐up (−41 ± 77%) values. HFDC also reduced diastolic blood pressure compared with values after LFDC (−6.7 ± 10.1 mmHg).

**Conclusions:**

Reductions in blood pressure and NT‐proBNP after HFDC indicate decreased vascular resistance resulting in reduced left ventricular afterload. These effects warrant further investigation in patients with chronic HF.

## Introduction

The prevalence of chronic heart failure (HF) or asymptomatic left ventricular (LV) dysfunction is estimated to be ≈4%; with between 10 and 20% of people aged over 75 years having some degree of HF.^1^ Ischaemic heart disease is the predominant cause of chronic HF.^1^, ^2^ Despite improvements in patient management through guideline‐directed medical therapy (GDMT), a progressive decline in cardiac function with high mortality is common.^1^, ^2^

Endothelial dysfunction, measured as decreased flow‐mediated dilatation (FMD), is closely linked to poor prognosis in chronic HF.^3^, ^4^, ^5^ Increased circulating levels of endothelin‐1 (ET‐1) or the proendothelin‐1 precursor fragment, C‐terminal proendothelin‐1 (CT‐proET‐1), are also predictive of mortality.^5^, ^6^, ^7^ This may reflect the degree of endothelial dysfunction or increased ET‐1 production by other tissues such as the myocardium.^8^ Age‐related development of endothelial dysfunction contributes to increased vascular resistance and raised blood pressure.^9^ These changes are likely important predisposing factors for the onset of symptoms of chronic HF in the elderly.^1^

Treatment of endothelial dysfunction in chronic HF represents an important area needing new therapies.^5^ High‐flavanol cocoa has potential as an adjunct to standard therapy as it increases endothelium‐dependent vasodilator responses in healthy individuals as well as in patients with diabetes and coronary artery disease (CAD).^10^, ^11^, ^12^ Flavanol‐rich dark chocolate also improves endothelial function in chronic HF.^13^ Whether such effects can provide a therapeutic benefit of sufficient magnitude in chronic HF to reduce morbidity and mortality has yet to be evaluated.

Here, in a pilot study, we investigated whether a high daily dose of cocoa flavanols could have therapeutic value in the treatment of chronic HF by using NT‐proBNP as a biomarker to assess changes in cardiac function after 4 week consumption of high‐flavanol dark chocolate (HFDC).

## Methods

### Study design and patients

The study was a single‐centre randomized double‐blind placebo‐controlled investigation with a crossover design. Ethical approval was obtained from the NHS Research Ethics Service (East London and the City Research Ethics Committee; reference number 07/Q0604/24). The study conformed to the principles outlined in the Declaration of Helsinki. Thirty‐two patients were recruited at the Heart Failure Clinic of the London Chest Hospital. All patients had documented evidence of previous ischaemic heart disease using invasive coronary angiography and/or non‐invasive testing. Patient criteria were as follows: (i) chronic HF due to LV systolic dysfunction confirmed by transthoracic echocardiography or left ventriculogram, with an NYHA functional classification of 2 or 3 (ejection fraction values were obtained from patient records), and (2) stable for at least 6 months on optimal GDMT, in accordance with the National Institute of Clinical Excellence guidelines for chronic HF 2003.^14^

The exclusion criteria were as follows: age <45 years; diabetes mellitus; LV dysfunction not related to systolic HF or ischaemic heart disease; exertional angina; atrial fibrillation; cardiac surgery, percutaneous coronary intervention, acute coronary syndrome, or stroke within 6 months of the study; active psychiatric or psychological illness; life expectancy <1 year because of unrelated disease; anticipated compliance issues; concurrent participation in other clinical investigations, whether active or in follow‐up; anaemia (Hb <10 g/dL); abnormal electrolytes or creatinine >200 µmol/L; anticoagulation therapy; chronic obstructive pulmonary disease; and treatment with aminophylline or theophylline.

Patient records were screened for eligibility. Potential participants were informed of the study. Those electing to participate gave written informed consent before baseline assessment and randomization to either HFDC (50 g/day) or placebo low‐flavanol dark chocolate (LFDC, 50 g/day) for 4 weeks. At the end of this period, patients were crossed over to receive the other chocolate for 4 weeks. Follow‐up assessment was undertaken 4 weeks after chocolate consumption had ceased. Four‐week intervals between measurements were used to ensure that any changes in plasma lipid profiles reached steady state,^15^ and because 4 week consumption of high‐flavanol cocoa produces a sustained improvement in endothelial function.^11^

### Dark chocolate products and randomization

High‐flavanol dark chocolate and LFDC of similar taste and appearance were manufactured by Barry Callebaut (Lebbeke‐Wieze, Belgium) (Table 1 shows relative compositions). Flavanol doses for LFDC (88 mg/day) and HFDC (1064 mg/day) were chosen based on previous studies showing that ≈80 mg of total flavanols/day was without significant effect on endothelial function, while large doses of ≈900 mg/day produced a consistent and marked increased in endothelium‐dependent flow‐mediated vasodilatation.^11^

**Table 1:** Composition of high‐flavanol and low‐flavanol dark chocolate bars

	HFDC	LFDC
Total cocoa solids (%)	65	65
Flavanols:		
Monomers, mg	223	23
Procyanidin dimers, mg	192	24
Procyanidin trimers to decamers, mg	649	41
Total flavanols (monomers to decamers), mg	1064	88
Theobromine, mg	395	420
Caffeine, mg	35	35
Total fat, g	19.9	18.8
Saturated, g	12.4	11.7
Monounsaturated, g	6.9	6.6
Polyunsaturated, g	0.6	0.5
Total protein, g	2.8	2.9
Available carbohydrates, g	18.2	18.8
Sugars, g	15.8	16.4
Starch, g	2.4	2.4
Dietary fibre, g	3.0	3.1
Calories, kcal	264	257

HFDC, high‐flavanol dark chocolate; LFDC, low‐flavanol dark chocolate.

Values are per 50 g bar (the daily amount consumed during the HFDC and LFDC phases of the study).

Chocolate was supplied as separate patient‐coded boxes for each phase of the study (50 g bars × 28/box, plain foil wrapped). Investigators were blinded to the randomization schedule. Patients were instructed to consume 25 g each morning and 25 g in the afternoon, to maintain their normal dietary habits, but not to drink red wine or take herbal medicines for HF, such as hawthorn extract.

### Patient examination and assessment

Patients underwent a physical examination with a 12‐lead ECG recording at baseline to ensure the absence of arrhythmias. Patient details and drug treatment were recorded. The following measurements were made at baseline and at 4, 8, and 12 weeks: NYHA assessment, body weight, blood pressure measured using an Omron BP Monitor 705CP II, and radial artery tonometry performed with pulse waveform analysis using the Sphygmocor Px Model and the accompanying software package. Blood samples were collected for measurement of NT‐proBNP, CT‐proET‐1, platelet function, high‐sensitivity C‐reactive protein (CRP), high‐sensitivity cardiac troponin I, theobromine, lipid profile (total cholesterol, LDL cholesterol, HDL cholesterol, and triglycerides), HbA1c, and standard haematological and biochemical parameters. Participants also completed a Quality of Life Questionnaire (SF‐36v2™ Health Survey © 1992–2002 from Health Assessment Lab, Medical Outcomes Trust and QualityMetric Incorporated).

Blood samples for measuring NT‐proBNP and CT‐proET‐1 were taken after patients had been at rest for at least 15 min into pre‐chilled EDTA tubes, placed on ice and centrifuged within 10 min using a cooled centrifuge (2000 g for 10 min at +4°C). Plasma was stored at −80°C until immunoassay. NT‐proBNP levels were measured by high‐sensitivity electrochemiluminescent sandwich immunoassay (Meso Scale Diagnostics, Gaithersburg, MD, USA). CT‐proET‐1 was measured by sandwich ELISA following published methods,^16^ with affinity‐purified sheep IgG specific for preproET‐1_[169–179]_ as the capture antibody and biotinylated affinity‐purified sheep IgG specific for preproET‐1_[204–212]_ as the detection antibody. CT‐proET‐1 assay standard was synthetic preproET‐1_[169–212]_ synthesized at the DBSB Core Facility, Faculty of Medicine, University of Geneva (Switzerland). High‐sensitivity cardiac troponin I was measured by sandwich ELISA (HyTest, Turku, Finland). CRP immunoassay was from MP Biomedicals (Orangeburg, NY, USA). Plasma theobromine and caffeine levels were measured by high‐performance liquid chromatography to confirm chocolate consumption.^17^

Platelet function was assessed using a Platelet Function Analyzer (PFA‐100^TM^; Dade Behring, West Sacramento, CA, USA), which measures shear stress‐induced platelet aggregation using both collagen–epinephrine and collagen–adenosine diphosphate (ADP) cartridges. Results are reported as closure times indicating the speed for blood to clot up to a maximum time of 300 s.^18^

### Statistical analyses

**Figure 1:**
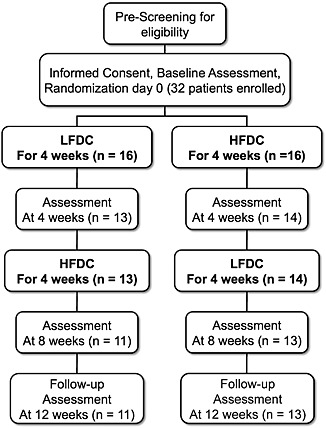
Flow diagram illustrating study design with the number of patients entering and completing each phase of the study. HFDC, high‐flavanol dark chocolate; LFDC, low‐flavanol dark chocolate.

Twenty‐four patients completed the study (Figure 1). However, only 20 patients had pulse waveform recordings of a sufficient quality index (>80%) at all time points for inclusion in the analyses of haemodynamic indices derived from pulse waveform‐derived measurements. The Quality of Life Questionnaire was completed by only 19 participants at every time point. Data were analysed with MiniTab or GraphPad Prism software. NT‐proBNP data were subjected to nonparametric analyses using the Friedman test followed by Wilcoxon signed‐rank test. Statistical differences for all other data were analysed by analysis of variance with *post hoc* comparison of differences between HFDC and the other time points using Fisher's least significant difference test. Patient data are reported as mean ± standard deviation unless otherwise indicated.

## Results

### Patient characteristics, study compliance, and tolerability

Thirty‐two patients were enrolled in the study, of which 24 completed all phases (Figure 1; patient characteristics are shown in Table 2). Ischaemic heart disease was the aetiology of HF in all patients. All patients were treated with an angiotensin‐converting enzyme inhibitor (ACE‐I) or an angiotensin receptor blocker and a beta‐blocker (except for one). Approximately half were treated with diuretics. The majority of patients were also taking aspirin and a statin.

**Table 2:** Baseline characteristics of patients completing the study

*n*	24
Age (years)	70 ± 10
Male gender	20 (80%)
Weight (kg)	85.7 ± 21.3
Body mass index (kg/m^2^)	29.6 ± 5.9
Systolic blood pressure (mmHg)	126.3 ± 17.8
Diastolic blood pressure (mmHg)	71.0 ± 9.8
Heart rate (bpm)	64.0 ± 6.8
NYHA chronic HF class II/III	20 (80%)/4 (20%)
LVEF (%)^a^	31.2 ± 9.9
Pacemaker	2 (8.3%)
Ethnicity	18 Caucasian (75%), 5 South Asian (20.8%), 1 Afro‐Caribbean (4.2%)
NT‐proBNP (pg/mL)	1970 ± 1412
Risk conditions for chronic HF, *n* (%):	
CAD/ischaemic	21 (87.5%)
Prior MI	19 (79.2%)
Previous PCI	10 (41.7%)
Previous CABG	4 (16.7%)
Hypertension	16 (66.7%)
Smoker	3 (12.5%) current, 8 (33.3%) ex‐smoker
Medication, *n* (%):	
ACE‐I/ARB	18/6 (100%)
ß‐blocker	23 (95.8%)
Diuretic	13 (54.2%)
Aldosterone blocker	7 (29.2%)
Aspirin	20 (83.3%)
Statin	21 (87.5%)
Nitrate	7 (29.2%)
Amlodipine	2 (8.3%)
Nicorandil	3 (12.5%)
Thyroxine	3 (12.5%)
Amiodarone	1 (4.2%)
Omacor	1 (4.2%)
Niacin	1 (4.2%)
Dipyridamole	1 (4.2%)

ACE‐I, angiotensin‐converting enzyme inhibitor; ARB, angiotensin receptor blocker; CABG, coronary artery bypass graft; CAD, coronary artery disease; HF, heart failure; LVEF, left ventricular ejection fraction; MI, myocardial infarction; NT‐proBNP, N‐terminal pro‐B‐type natriuretic peptide; NYHA, New York Heart Association; PCI, percutaneous coronary intervention.

Data are presented as mean ± standard deviation or number (per cent).

a There were no ejection fraction values for two patients with poor echogenic windows that prevented accurate quantification.

Eight of the 32 patients initially enrolled dropped out for a variety of reasons (withdrawals: up to 4 weeks—unpalatable taste of chocolate, LFDC *n* = 1, and HFDC *n* = 2; other LFDC *n* = 2, one fall, and one unscheduled gastrointestinal surgery; and 4–8 weeks, LFDC *n* = 1 conflicting personal commitments, HFDC *n* = 2, one episode of vomiting after 2 weeks of HFDC, and one planned surgery). Several patients reported mild gastrointestinal side effects while consuming dark chocolate including loose stools or diarrhoea (LFDC *n* = 2; HFDC *n* = 6). Four patients who completed the study complained about the taste of the test products and found it difficult to eat 50 g per day. One patient had swollen ankles during the first 4 weeks while consuming LFDC and had her dose of diuretic increased by her GP. One patient reported paraesthesia in the right hand after 4 weeks of HFDC. One patient had an episode of troponin‐negative chest pain while consuming HFDC. Theobromine levels were increased to a similar degree after HFDC and LFDC compared with baseline and follow‐up values (Table 3), confirming consumption of chocolate over the two 4 week periods. Background levels of theobromine at baseline and follow‐up are likely due to caffeinated drink consumption as theobromine is also a metabolite of caffeine. Caffeine levels did not alter during the study.

**Table 3:** Effects of 4 week consumption of LFDC and HFDC

	Baseline	LFDC	HFDC	Follow‐up
Methylxanthines				
Theobromine (mg/L)	1.79 ± 1.24	5.74 ± 3.13*	6.70 ± 3.98*	1.78 ± 1.18
Caffeine (mg/L)	2.68 ± 2.19	2.32 ± 1.00	2.57 ± 1.48	2.21 ± 1.29
Biomarker measurements				
CT‐proET‐1 (pmol/L)	16.3 ± 3.7	16.2 ± 3.6	16.8 ± 3.7	16.6 ± 3.9
CRP (mg/L)	4.05 ± 3.60	4.39 ± 3.63	7.13 ± 8.94	4.92 ± 4.24
Cardiac troponin I (ng/L)	3.06 ± 2.65	3.06 ± 2.49	2.89 ± 1.91	2.93 ± 2.17
Platelet function (PFA‐100, stimulated closure times)				
ADP (s)	75.6 ± 18.5	76.1 ± 12.4	73.8 ± 16.8	84.0 ± 49.1
Epinephrine (s)^a^	182.7 ± 74.9	177.1 ± 68.9	169.3 ± 83.3	176.2 ± 77.3
Haematological and biochemical analyses				
Total cholesterol (mmol/L)	4.25 ± 1.17	4.28 ± 1.26	4.08 ± 0.80	4.22 ± 1.15
LDL cholesterol (mmol/L)	2.14 ± 1.18	2.20 ± 1.04	1.97 ± 0.65	2.15 ± 1.08
HDL cholesterol (mmol/L)	1.29 ± 0.33	1.35 ± 0.37	1.35 ± 0.43	1.30 ± 0.36
Triglycerides (mmol/L)	1.75 ± 0.87	1.70 ± 0.79	1.68 ± 0.77	1.66 ± 0.76
HbA1c %	6.03 ± 0.46	6.05 ± 0.52	5.98 ± 0.49	6.01 ± 0.51
Na^+^ (mmol/L)	142.2 ± 2.3	141.7 ± 2.5	141.6 ± 2.3	141.5 ± 2.9
K^+^ (mmol/L)	4.48 ± 0.38	4.57 ± 0.43	4.63 ± 0.41	4.46 ± 0.39
Urea (mmol/L)	8.55 ± 3.06	8.70 ± 3.94	8.80 ± 3.99	8.59 ± 2.95
Creatinine (µmol/L)	104.7 ± 27.7	102.3 ± 28.6	104.3 ± 27.6	101.9 ± 30.0
Alanine transaminase (IU/L)	23.8 ± 14.3	24.5 ± 13.3	21.7 ± 8.1	21.3 ± 6.2
Hb (g/dL)	13.3 ± 1.6	13.2 ± 1.6	13.1 ± 1.4	13.1 ± 1.4
MCV (fL)	89.1 ± 5.8	88.7 ± 5.3	88.4 ± 5.1	88.6 ± 5.6
WBC 10^−9^/L	7.78 ± 1.45	7.33 ± 1.59	7.92 ± 1.42	7.65 ± 1.60
Platelets 10^−9^/L	243 ± 51	243 ± 62	255 ± 70	247 ± 63
Quality of Life Questionnaire^b^				
Physical well‐being score	36.6 ± 10.1	36.8 ± 10.0	37.6 ± 10.9	36.9 ± 10.7
Mental well‐being score	45.9 ± 14.2	46.8 ± 8.8	46.9 ± 10.3	45.7 ± 11.8
Body weight (kg)	85.7 ± 21.3	86.1 ± 20.8	86.1 ± 20.6	85.6 ± 21.1

ADP, adenosine diphosphate; CRP, C‐reactive protein; CT‐proET‐1, C‐terminal proendothelin‐1; HFDC, high‐flavanol dark chocolate; LFDC, low‐flavanol dark chocolate; MCV, mean cell volume; WBC, white blood cell count.

One‐way analysis of variance with Fisher's least significant difference test:

* *P* < 0.001 compared with baseline or follow‐up.

a Closure times exceeding 300 s were recorded as a closure time of 300 s for statistical analyses.

b *n* = 19 for study participants completing the questionnaire at all time points.

### Changes in N‐terminal pro‐B‐type natriuretic peptide

**Figure 2:**
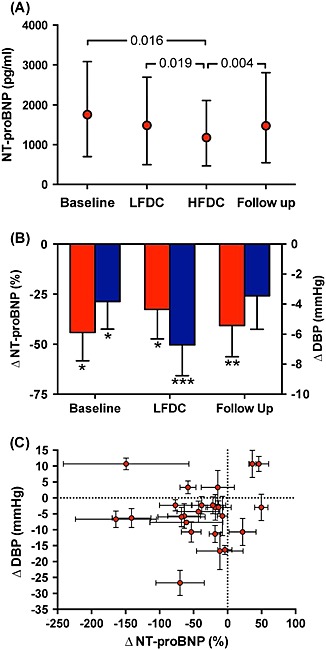
(A) Plasma measurements of N‐terminal pro‐B‐type natriuretic peptide (NT‐proBNP) at baseline, after 4 weeks of low‐flavanol dark chocolate (LFDC), after 4 weeks of high‐flavanol dark chocolate (HFDC), and at follow‐up (median with 25th and 75th percentiles). Friedman test, *P* = 0.017 for the effect of HFDC (individual *P*‐values derived from Wilcoxon signed rank test). (B) Changes in NT‐proBNP (%) (red bars) and diastolic blood pressure (DBP) (mmHg) (blue bars) after HFDC compared with values at baseline, after LFDC, and at follow‐up (mean ± SEM; *, **, *** = *P* <0.05, <0.01, <0.001, respectively). (C) Individual patient changes in NT‐proBNP (%) plotted relative to changes in DBP (mmHg), differences (mean ± SEM) after HFDC compared with values at baseline, after LFDC, and at follow‐up.

Plasma NT‐proBNP was significantly lower after HFDC compared with baseline (−44 ± 69%, *P* = 0.016), LFDC (−33 ± 72%, *P* = 0.019), and follow‐up values 4 weeks after completion of chocolate consumption (−41 ± 77%, *P* = 0.004) (Figure 2). The corresponding changes expressed as median decreases (with interquartile range) were −271 (871), −178 (854), and −245 (744) pg/mL. The overall mean reduction in NT‐proBNP after HFDC compared with baseline, LFDC, and follow‐up values was −39 ± 56%, with 12 of the 24 patients showing an average decrease ≥30% (*Figure* 2).

### Changes in blood pressure

After 4 week consumption of HFDC, brachial artery diastolic blood pressure (DBP) was decreased compared with measurements obtained after LFDC (−6.7 ± 10.1 mmHg, *P* = <0.001) and at baseline (−3.8 ± 9.0 mmHg, *P* = 0.045) (Table 4, *Figure* 2 *B*). For each patient, the mean decrease in DBP after HFDC compared with baseline, LFDC, and follow‐up was compared to mean reductions in NT‐proBNP (Figure 2C). Although the majority of subjects showed an overall mean decrease in both DBP and NT‐proBNP after HFDC compared with the other time points, these changes were not correlated (*r* = 0.09; *P* = 0.67). Central DBP derived from the peripheral pressure waveform was also reduced after HFDC compared with LFDC (−7.0 ± 10.7 mmHg, *P* = 0.002) (Table 4). Central and peripheral systolic blood pressure (SBP) did not alter significantly during the study. Central and peripheral pulse pressures tended to increase after HFDC, but these changes did not reach significance. Other haemodynamic indices derived from pulse waveform analyses did not show significant differences.

**Table 4:** Blood pressure measurements

Brachial artery (*n* = 24):				
	Baseline	LFDC	HFDC	Follow‐up
SBP	126.3 ± 17.8	124.9 ± 15.7	123.9 ± 22.5	126.3 ± 19.1
DBP*	71.0 ± 9.8	73.8 ± 10.4	67.1 ± 10.5[Link], b	70.6 ± 10.9
PP	55.3 ± 13.0	51.0 ± 14.9	56.8 ± 20.0	55.7 ± 15.7
MBP	89.4 ± 11.5	90.8 ± 10.3	86.0 ± 12.4	89.2 ± 12.1
HR	64.0 ± 6.8	61.3 ± 9.5	63.9 ± 7.7	63.8 ± 8.6
Derived from pulse wave analyses (*n* = 20):				
cSBP	117.1 ± 17.5	116.2 ± 16.3	115.3 ± 23.5	117.8 ± 20.3
cDBP**	71.7 ± 11.0	74.6 ± 11.3	67.5 ± 11.6^c^	70.7 ± 12.1
cPP	45.4 ± 10.9	41.7 ± 14.8	47.8 ± 18.8	47.5 ± 14.7
cMBP	89.7 ± 13.2	90.5 ± 11.8	86.0 ± 15.0	88.9 ± 15.0
AIx	30.2 ± 9.4	28.4 ± 10.3	29.5 ± 12.3	30.2 ± 12.9

c, central; DBP, diastolic blood pressure; HFDC, high‐flavanol dark chocolate; HR, heart rate; LFDC, low‐flavanol dark chocolate; MBP, mean blood pressure; PP, pulse pressure; SBP, systolic blood pressure.

Values BP (mmHg), HR (bpm), augmentation index (AIx, %), mean ± standard deviation.

Analysis of variance:

* *P* = 0.008,

** *P* = 0.016.

Fisher's least significant difference test:

a Baseline versus HFDC, *P* = 0.045,

b LFDC versus HFDC, *P* = <0.001,

c LFDC versus HFDC, *P* = 0.002.

### Effect on platelet function

No change in platelet function was detected using the PFA‐100^TM^ analyzer. Low‐dose aspirin has little or no effect on platelet ADP responses measured with the PFA‐100^TM^ analyzer, whereas complete suppression of epinephrine responses, seen as non‐closure (>300 s), is observed in approximately 50% of the subjects.^18^ Here, 20 out of the 24 patients were treated with low‐dose aspirin. Approximately 50% of aspirin‐treated subjects exhibited non‐closure with epinephrine at some point in the study. HFDC did not increase the frequency of non‐closure with epinephrine and had no effect on ADP responses (Table 3).

### Other measurements

No changes in circulating levels of CT‐proET‐1, CRP, or cardiac troponin I were observed after HFDC compared with baseline, LFDC, or follow‐up values (Table 3). None of the haematological or biochemical parameters that were assessed showed changes in response to either LFDC or HFDC (Table 3). For the 19 patients completing the Quality of Life Questionnaire at every clinic visit, there were no changes in overall physical or mental well‐being scores or in the individual component scores (Table 3). There was a trend for increased body weight during consumption of dark chocolate (LFDC and HFDC) (*P* = 0.4, Table 3).

## Discussion

In this randomized crossover pilot study of patients with chronic HF due to systolic LV dysfunction and prior history of ischaemic heart disease, HFDC decreased NT‐pro‐BNP levels and DBP. These findings indicate decreased peripheral vascular resistance with a consequent reduction in cardiac afterload and suggest that potential improvements in cardiac function after consumption of flavanols warrant further exploration.

### Clinical relevance of the findings

Vasodilator therapy is a key component of GDMT in chronic HF.^1^, ^2^ However, hypotension, as a consequence of excessive vasodilatation, is a frequent dose‐limiting side effect. Previous patient studies of high‐flavanol cocoa and dark chocolate have reported reproducible improvements in endothelial function with similar or somewhat lower daily amounts of flavanols than used here,^11^, ^12^, ^13^ but the long‐term impact of improving endothelium‐dependent vasodilatation has yet to be determined. Here, in patients with chronic HF whose treatment was considered fully optimized according to current guidelines, daily consumption of a high dose of cocoa flavanols reduced NT‐proBNP. Consistent with a mechanism of action leading to improved endothelial function and peripheral vasodilatation, DBP was also reduced in this study. In comparison, SBP did not decrease significantly, and pulse pressure tended to increase, indicating that cardiac output was increased after HFDC. Hence, lower NT‐proBNP probably reflects reduced LV afterload because of decreased peripheral vascular resistance.

No changes were observed in NYHA classification or in the quality of life assessment of changes in physical, mental, and social functioning. But the treatment period with HFDC is likely too short to achieve quantifiable changes in these measures.

### Prognostic significance of reductions in N‐terminal pro‐B‐type natriuretic peptide

Plasma levels of BNP and NT‐proBNP are sensitive biomarkers of the severity of chronic HF, which predict prognosis.^19^ Treatment strategies aimed specifically at reducing NT‐proBNP result in fewer cardiovascular events.^20^ Reductions in BNP or NT‐proBNP are associated with lower mortality, with decreases ≥30% associated with less adverse outcomes.^21^ NT‐proBNP decreased by >30% in half the patients here, so adding cocoa flavanols to GDMT has potential to reduce mortality compared with standard therapy alone.

### Cocoa flavanols as a treatment for endothelial dysfunction

Despite evidence that endothelial dysfunction is linked to poor prognosis in chronic HF, there are no treatments that specifically reverse this decline in vasodilator function.^5^ ACE‐Is and statins produce modest improvements in endothelial function.^5^, ^22^ In comparison, cocoa flavanols increase FMD responses in patients with diabetes, CAD, or chronic HF even when concurrent treatment includes these medications.^11^, ^12^, ^13^ Here, all patients were treated with an ACE‐I or angiotensin receptor blocker, and the majority also received statins, so flavanol‐induced changes in vascular function with reductions in NT‐proBNP are additive to that achieved by GDMT.

**Figure 3:**
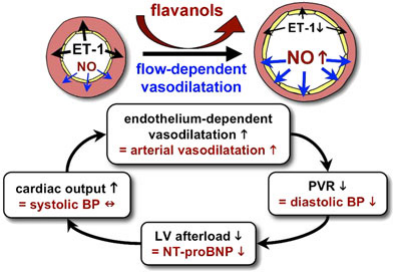
Schematic hypothesis for how flavanols reduce N‐terminal pro‐B‐type natriuretic peptide (NT‐proBNP) levels and improve cardiac function in chronic heart failure. Flavanols increase nitric oxide synthesis, suppress endothelin‐1 (ET‐1) synthesis, and restore endothelium‐mediated flow‐dependent vasodilatation. LV, left ventricular; PVR, peripheral vascular resistance.

Endothelial dysfunction is both a cause and consequence of HF. Flow‐dependent vasodilatation is mediated by the endothelium in response to shear stresses exerted by flowing blood on the vascular wall. Indeed, reduced reactive hyperaemia is associated with low basal forearm blood flow, likely as a result of low cardiac output.^4^ The importance of this as a factor underlying decreased FMD responses in chronic HF is highlighted by observations that heart transplantation can reverse endothelium‐dependent vasodilator responses.^23^, ^24^ Current evidence indicates that flavanols restore flow‐mediated vasodilatation. In chronic HF, this may result in reduced peripheral vascular resistance and lower DBP, with a consequent reduction in LV afterload leading to decreased myocardial release of NT‐proBNP. We hypothesize that flavanol‐induced peripheral vasodilatation enables cardiac output to increase, so that SBP is maintained, which in turn further improves flow‐mediated vasodilatation (Figure 3).

Plasma levels of ET‐1 and the relatively stable big ET‐1 and CT‐proET‐1 (a fragment of the ET‐1 precursor) are increased in HF and predict outcome.^5^, ^6^, ^7^ Changes in plasma biomarkers of ET‐1 synthesis may be expected to indicate endothelial dysfunction. Cocoa flavanols inhibit ET‐1 synthesis by endothelial cells (see Supporting Information Appendix S1 comparing the effects of flavanol extracts prepared from HFDC and LFDC);^25^ nevertheless, HFDC had no effect on circulating CT‐proET‐1 levels. However, the main source of plasma CT‐proET‐1 in chronic HF may be the myocardium, or other vascular cells, rather than the endothelium,^8^ hence limiting the potential for CT‐proET‐1 as a biomarker of endothelial function. Studies of other endothelial biomarkers (e.g. von Willebrand factor and E‐selectin) also showed no relationship with BNP.^5^

### Effect of flavanols on platelet function

Chronic HF increases platelet thrombogenicity and the risk of thrombotic events.^26^ Flavanols have been reported to have anti‐platelet activity.^13^ In a previous study in chronic HF, flavanol‐rich chocolate caused an acute reduction in shear stress‐dependent platelet adhesion, but after 4 week consumption, this response was lost.^13^ Similarly, no change in platelet function was observed here after 4 weeks of HFDC.

### Optimal daily amount of flavanols in patients with chronic heart failure

Improvements in endothelial function after cocoa flavanols are dose related.^11^ In patients with type 2 diabetes (>70% with a history of CAD), FMD was markedly increased after a single cocoa drink (963 mg of flavanols),^11^ whereas the response to 371 mg of flavanols was substantially less and 75 mg had no effect.^11^ Daily treatment of diabetic patients for 1 month (321 mg thrice daily) increased both basal FMD and the acute response measured 2 h after a morning cocoa drink (321 mg of flavanols).^11^ Similarly, 750 mg of flavanols/day improved FMD responses and also decreased SBP patients with CAD,^12^ but <500 mg/day is likely insufficient as endothelial function did not improve after 6 weeks in hypercholesterolemic women (446 mg of flavanols/day)^27^ or in another study of patients with established CAD (444 mg daily).^28^

In chronic HF, a flavanol‐rich chocolate (624 mg of flavanols/day for 4 weeks) increased FMD responses but did not change BP or NT‐proBNP.^13^ Therefore, a higher daily amount may be required for a clinically significant improvement in cardiac function. Whether 1064 mg of flavanols/day, the amount used here, is optimal in chronic HF requires confirmation in future dose‐ranging studies.

### Composition of cocoa flavanols and relevance to future clinical trials

Cocoa flavanols consist of a mixture of monomers (such as (−)‐epicatechin), dimers, and oligomeric procyanidins (OPC), which are mainly oligomers of (−)‐epicatechin ranging from trimers to decamers.^29^ Schroeter and colleagues attributed the increase in FMD observed in healthy volunteers after a high‐flavanol cocoa drink to (−)‐epicatechin.^10^ However, purified (−)‐epicatechin produced only 30% of the response seen with cocoa flavanols,^10^ indicating that OPC play a role in this vascular effect. Here, OPC represented ≈60% of total cocoa flavanols in HFDC (Table 1). Experimentally, OPC have potent actions on endothelial function inducing endothelium‐dependent vasodilatation and inhibiting ET‐1 synthesis.^25^, ^30^, ^31^ Currently, little is known about the fate of OPC following oral administration. Until it is fully understood which components of cocoa flavanols are responsible for these effects on vascular function, it will be difficult to optimize products for medical use.

Interestingly, hawthorn extract (*Cretageus* species), a traditional remedy for chronic HF, contains flavanols mainly as OPC.^30^ WS 1442 is a hawthorn extract composed of 18.75% OPC, which causes endothelium‐dependent vasodilatation of isolated coronary arteries.^32^ Several clinical trials in chronic HF have evaluated WS 1442. A dose of 1800 mg/day (337 mg OPC per day) improved exercise tolerance, but half of this dose lacked significant functional effects.^33^ The lower dose (900 mg WS 1442 per day; ≈170 mg OPC) was used for the SPICE trial, a multi‐centre trial in 2681 patients with chronic HF.^34^ At this dose, patients with an LVEF ≥25–35% had a 39% reduction in sudden death, but there was no change in total mortality. Effects on NT‐proBNP were not reported.^34^ The same dose of WS 1442 was also used in the Hawthorn Extract Randomized Blinded Chronic Heart Failure trial. This showed no effect on 6 min walking distance, blood pressure, or BNP.^35^ Although some have concluded that hawthorn extract has no beneficial effects in patients optimized to GDMT, the lack of efficacy in these trials can also be explained by the use of an ineffective dose of WS 1442 (≈170 mg OPC),^34^, ^35^ compared with HFDC (649 mg OPC) used here.

### Strengths and limitations

A key strength of this study is that patients were stable on GDMT prior to enrolment so that NT‐proBNP levels were fairly constant throughout the study. The crossover design strengthens the conclusion that the reduction in NT‐proBNP levels after HFDC compared with all other time points was due to the flavanol components. However, the absence of a pretreatment period to assess baseline fluctuations in NT‐proBNP prior to randomisation to HFDC or LFDC is a weakness of the study design. Further limitations include the small sample size, the short period of investigation, and the absence of FMD measurements or assessment of LV function to correlate with blood pressure and reductions in NT‐proBNP. In addition, all patients had a history of ischaemic heart disease, which limits the generalizability of the findings relative to the broader spectrum of patients with chronic HF of different aetiologies.

## Conclusions

This study indicates that combining cocoa flavanols with GDMT has potential for improving cardiac function in chronic HF. Dark chocolate may not be the ideal product because of its poor palatability and high calorie content. However, optimisation of a flavanol supplement requires identification of the principal active components in mixed flavanol extracts that are responsible for changes in FMD, blood pressure, and NT‐proBNP. Future studies should also determine whether the effect on NT‐proBNP is sustained and correlated with improvements in LV function and exercise capacity.

## Funding

This work was supported by a grant (to R.C.) from Barry Callebaut Belgium NV. Assay of CT‐proET‐1 was supported by a grant from the Medical Research Council, UK (grant number G0801509).

## Conflicts of interest

R.C. is a director of FlavoSanté Ltd. All other authors have no conflict of interest to declare.

## Supporting information


**Appendix S1.** Bioassay of extracts of the test chocolates.
**Figure S1.** Inhibition of ET‐1 synthesis by cultured endothelial cells with chocolate flavanol extracts prepared from HFDC and LFDC. Extract concentrations expressed equivalent to weight of chocolate samples. Results are mean ± SEM for extracts of two samples of each chocolate evaluated in triplicate in two experiments.

Supporting info item

Supporting info item
