# Supplementary figures and images for: Cocoa flavanols reduce N‐terminal pro‐B‐type natriuretic peptide in patients with chronic heart failure
Source: ESC Heart Fail. 2015 Dec 8;3(2):97–106. doi: 10.1002/ehf2.12077 (PMC4985700; doi:10.1002/ehf2.12077)

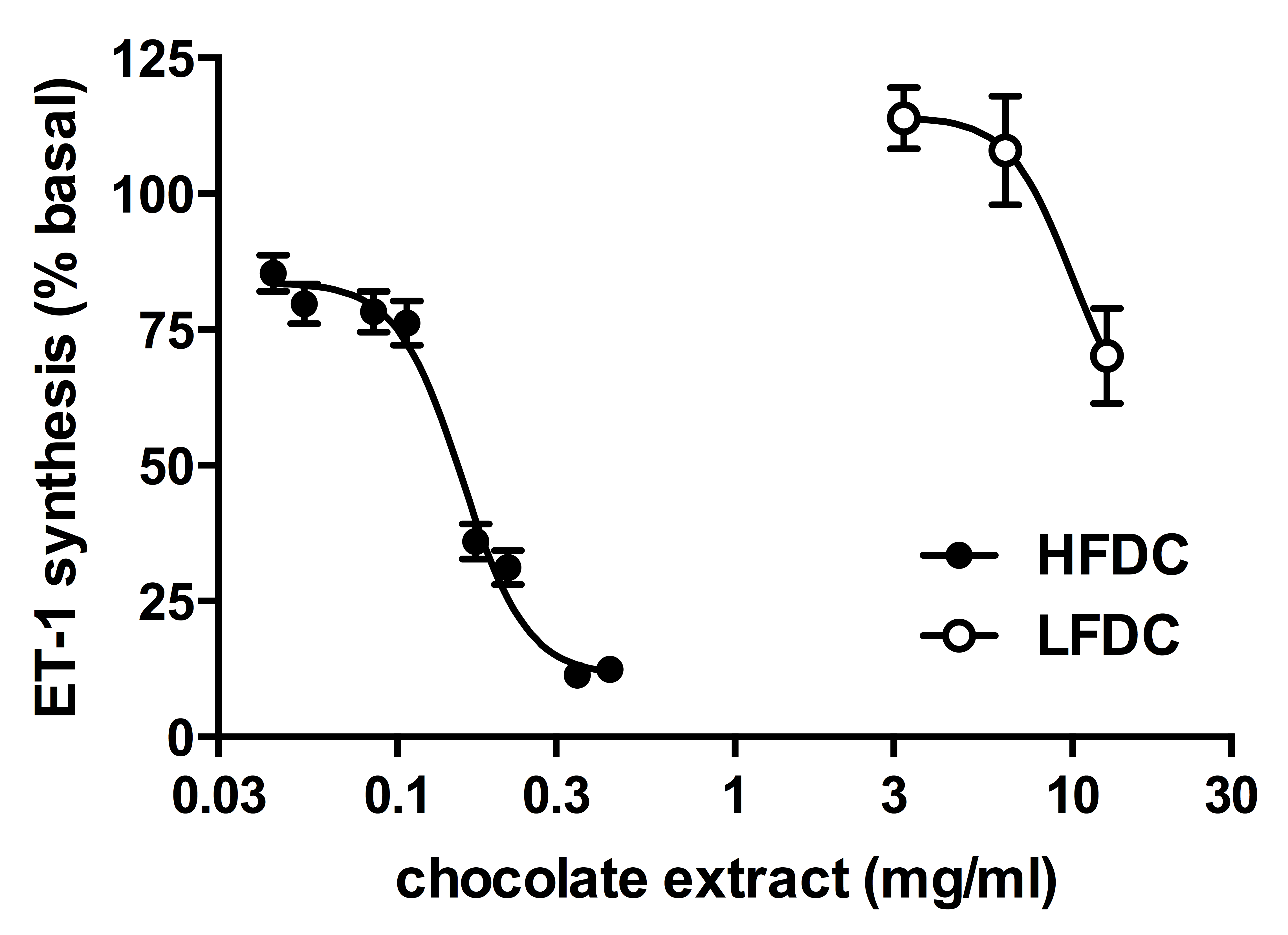

Supplement: Supplementary file 1 — Supporting info item [file EHF2-3-097-s001.tiff]
