# Supplementary material for: Cocoa flavanols reduce N‐terminal pro‐B‐type natriuretic peptide in patients with chronic heart failure
Source: ESC Heart Fail. 2015 Dec 8;3(2):97–106. doi: 10.1002/ehf2.12077 (PMC4985700; doi:10.1002/ehf2.12077)
Supplement: Supplementary file 2 — Supporting info item [file EHF2-3-097-s002.docx]

**APPENDIX S1**

**Cocoa Flavanols Reduce N-terminal pro–B-Type Natriuretic Peptide in Patients with Chronic Heart Failure**

Rodney De Palma, Imelda Sotto, Elizabeth G. Wood, Noorafza Q. Khan, Jane Butler, Atholl Johnston, Martin T. Rothman, and Roger Corder

**BIOASSAY OF EXTRACTS OF THE TEST CHOCOLATES**

High flavanol dark chocolate (HFDC) was manufactured by the Acticoa^TM^ method to preserve naturally high level of flavanols in raw cocoa beans through to fully processed chocolate. LFDC was manufactured from cocoa mass containing low levels of flavanols. The relative effectiveness of the two chocolates to modify endothelial function was evaluated by preparing defatted extracts and comparing the degree of inhibition of endothelin-1 **(**ET-1) synthesis by cultured bovine aortic endothelial cells (BAEC) following previously described methods.^1-3^ Based on inhibition of ET-1 synthesis, BAEC have comparable sensitivity to human aortic endothelial cells (HAEC) in terms of response to flavanol extracts (particularly procyanidins).^1^ However, BAEC have the advantage of higher ET-1 synthesis with more reproducible and more robust growth in cell culture compared to HAEC, making BAEC more suitable for screening biological activity of a large number of samples.^2^

**Methods**

Representative samples (weighing 1 – 2 g) of HFDC and LFDC (2 samples of each chocolate) were defatted with hexane, and extracted in 90% methanol/0.1% formic acid. Sample extracts were diluted in 0.1% formic acid and concentrated on columns of Sephadex LH-20.^3^ After rinsing with 30% methanol/0.2% formic acid, flavanols were eluted with 70% acetone, and dried down with a stream of N_2_.^2,3^ Extracts of cocoa flavanols were reconstituted in ultra pure H_2_O and stored frozen at -20ºC until bioassay on cultured BAEC.^1-3^ ET-1 synthesis was measured as previously described.^1-3^ Results are expressed relative to the weight of chocolate per ml of culture media, and analysed using GraphPad Prism software (San Diego, CA, USA).

**Results**

Extracts of HFDC (1064 mg total flavanols/50 g bar) produced a concentration dependent inhibition of ET-1 synthesis by endothelial cells (IC_50_ ≈ 0.15 mg HFDC/ml or ≈ 3.2 µg flavanols/ml) (Appendix Figure 1). This is comparable to previously published data with a high flavanol cocoa extract.^3^ In comparison, LFDC contained ≈ 8% the amounts of flavanols (Table 1), and the relative inhibition of ET-1 synthesis with LFDC extracts was <1% of the effect of HFDC (Figure S1). The greater degree of inhibition of ET-1 synthesis with extracts of cocoa flavanols prepared from HFDC is consistent with the high OPC content of this product (Table 1).

**References**

1. Corder R, Warburton RC, Khan NQ, Brown RE, Wood EG, Lees DM. The procyanidin-induced pseudo laminar shear stress response: new concept for the reversal of endothelial dysfunction. *Clin Sci* 2004;**107**:513-517.

2. Corder R, Mullen W, Khan NQ, Marks SC, Wood EG, Carrier MJ, Crozier A. Oenology: red wine procyanidins and vascular health. *Nature* 2006;**444**:566.

3. Caton PW, Pothecary MR, Lees DM, Khan NQ, Wood EG, Shoji T, Kanda T, Rull G, Corder R. Regulation of vascular endothelial function by procyanidin-rich foods and beverages. *J Agric Food Chem* 2010;**58**:4008-4013.
